# Heparin Mimetics: Their Therapeutic Potential

**DOI:** 10.3390/ph10040078

**Published:** 2017-10-02

**Authors:** Shifaza Mohamed, Deirdre R. Coombe

**Affiliations:** 1 School of Biomedical Sciences, Curtin Health Innovation Research Institute, Faculty of Health Sciences, Curtin University, Perth 6102, Western Australia; Shifaza.Mohamed@curtin.edu.au; 2 School of Applied Chemistry, Faculty of Science and Engineering, Curtin University, Perth 6102, Western Australia

**Keywords:** heparin mimetics, heparin, heparan sulfate, glycosaminoglycan, anticoagulant, cancer, anti-inflammatory

## Abstract

Heparin mimetics are synthetic and semi-synthetic compounds that are highly sulfated, structurally distinct analogues of glycosaminoglycans. These mimetics are often rationally designed to increase potency and binding selectivity towards specific proteins involved in disease manifestations. Some of the major therapeutic arenas towards which heparin mimetics are targeted include: coagulation and thrombosis, cancers, and inflammatory diseases. Although Fondaparinux, a rationally designed heparin mimetic, is now approved for prophylaxis and treatment of venous thromboembolism, the search for novel anticoagulant heparin mimetics with increased affinity and fewer side effects remains a subject of research. However, increasingly, research is focusing on the non-anticoagulant activities of these molecules. Heparin mimetics have potential as anti-cancer agents due to their ability to: (1) inhibit heparanase, an endoglycosidase which facilitates the spread of tumor cells; and (2) inhibit angiogenesis by binding to growth factors. The heparin mimetic, PI-88 is in clinical trials for post-surgical hepatocellular carcinoma and advanced melanoma. The anti-inflammatory properties of heparin mimetics have primarily been attributed to their ability to interact with: complement system proteins, selectins and chemokines; each of which function differently to facilitate inflammation. The efficacy of low/non-anticoagulant heparin mimetics in animal models of different inflammatory diseases has been demonstrated. These findings, plus clinical data that indicates heparin has anti-inflammatory activity, will raise the momentum for developing heparin mimetics as a new class of therapeutic agent for inflammatory diseases.

## 1. Introduction

**Figure 1:**
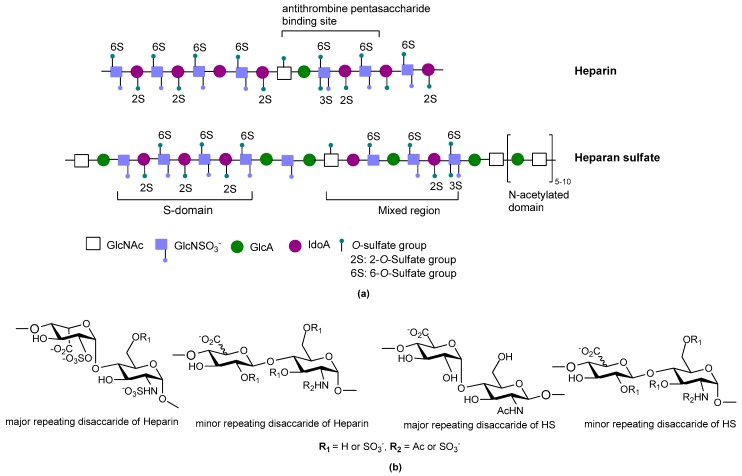
(**a**) Cartoon illustration of heparin and heparan sulfate structure; (**b**) Major and minor disaccharide repeating units in heparin and heparan sulfate.

Sulfated glycosaminoglycans (GAGs) are glycans present on mammalian cell surfaces and in the extracellular matrix (ECM). They are synthesized covalently attached to their specific core proteins and the resulting proteoglycans may be transmembrane, linked to the membrane by a glycosylphosphatidylinositol anchor, or they may be secreted and comprise an integral part of the ECM. The GAG chains are responsible for much of the activities of proteoglycans as they can bind selectively to a variety of proteins and pathogens making them very relevant to many disease processes, such as inflammation [1], angiogenesis [2], neurodegeneration [3], cardiovascular disorders [4], cancers [5] and infectious diseases [6]. Heparin is perhaps the best known member of the GAG family. It is a highly negatively-charged, linear polysaccharide found in the secretory granules of connective tissue mast cells where it is the GAG chains of the proteoglycan, serglycin [7]. Heparin/heparan sulfate (HS) are composed of 1-4 linked repeating disaccharide units comprising a uronic acid (d-glucuronic acid (GlcA) or l-iduronic acid (IdoA)) and d-glucosamine (GlcN) (Figure 1). Heparin and HS chains are remarkably heterogeneous as during biosynthesis they are modified by epimerization of GlcA to IdoA, and by sulfation at different positions on mainly GlcN and IdoA residues. HS contains a greater proportion of GlcA, whereas heparin contains more IdoA. Heparin, with its high content of sulfo and carboxyl groups, is a polyelectrolyte, having the highest negative charge density of any known biological macromolecule [8].

Heparin is often said to have been discovered in 1916 by Jay McLean, a medical student of Johns Hopkin Medical School, to whom has also been attributed the discovery of heparin’s anticoagulant effects in in vitro experiments [9]. However, the first descriptions of heparin as an anticoagulant were a little earlier and are summarized in a 1912 publication by Maurice Doyon [10]. In the 1930s clinical trials were conducted for heparin as an anticoagulant. Since then heparin has been used as a major clinical anticoagulant for many decades. However, its precise chemical structure, the range of its biological activities, and its Structure Activity Relationships (SAR) are not fully understood. Although biological functional studies of heparin were initially focused on its anticoagulant properties, in recent years the extent of its involvement in a range of other fundamental biological processes essential for normal mammalian development and physiology has been recognized. As such, heparin binds a multitude of proteins in addition to antithrombin III, giving rise to its well-appreciated polypharmacy [10].

For over 50 years, heparin gained widespread popularity and was extensively used in clinical practice. However, low-molecular-weight-heparin (LMWH) preparations have largely replaced heparin for clinical use over the past decade. This can be attributed to their superior pharmacokinetic profiles and their potential use in a wider range of clinical applications. However, LMWHs are not entirely free from the clinical disadvantages of its parent drug [11]. Heparin preparations have the potential to cause heparin-induced thrombocytopaenia type 2 which is an immunological response that involves generation of antibodies against a heparin-platelet factor 4 complex. Furthermore, non-specific binding to clotting factors by long chain heparin is believed to contribute to hemorrhagic side effects of the drug. Another disadvantage of heparin therapy is that its size and charge make parenteral administration a necessity.

In its natural state, heparin is a heterogeneous mixture of polysaccharide chains of different lengths and with different sulfation patterns; some of these chains are up to 100 saccharides in length [12]. Over the years there has been considerable debate as to whether distinct heparin sulfation motifs are required for the binding of different proteins. The current consensus is that the degree of structural specificity of motifs within heparin for protein binding is dependent upon the particular protein. Some of these interactions are highly specific and require the rare 3-*O*-sulfate group, whereas most proteins use *N*- and 2-*O*-sulfates, which in heparin are extremely common [13]; the most abundant disaccharide unit being a 2-*O*-sulfated iduronate linked to an *N*- and 6-*O*-sulfated glucosamine [14]. Importantly, the fact that heparin structures can bind a particular protein does not imply that the structure can necessarily modulate that protein’s function. Modulation of a protein’s function can depend on (1) where the heparin structure binds the protein; or (2) whether binding is of sufficient affinity to trigger a conformational change in the protein, as is the case when antithrombin III binds the pentasaccharide sequence from heparin containing the rare 3-*O*-sulfated glucosamine. In relation to the first point we have shown that a number of structurally different heparin tetrasaccharides can bind the chemokine, CCL5, but only those tetrasaccharides with a particular sulfation pattern preferentially bound CCL5 at a site that interfered with its ability to bind its cell surface receptor [15]. Consequently, success with developing heparin inspired therapeutic agents relies not only on identifying heparin-like structures, or heparin mimetics, that bind the protein of interest, but also in determining the effects of these structures on the biological functions of the protein target.

Heparin mimetics are synthetic or semi-synthetic compounds that are highly sulfated (usually), anionic, structurally distinct analogues of GAGs. Compounds (not other types of GAGs) that perform similar functions as heparin such as binding to the heparin-binding site on a protein may also be characterized as a heparin mimetic. Despite the increased understanding of the complex structure of heparin, the synthesis of clinically useful heparin mimetics is a relatively recent achievement.

The rational for development of heparin mimetics are numerous and varied, but a key consideration is that the mimetics are expected to overcome many of the problems associated with the parent molecule. The prospect of developing mimetics that display a higher relative potency and greater selectivity of action than its parent molecule is a major driving factor in this field of research. The need to curtail (but not necessarily eliminate) heparin’s polypharmacy and its anticoagulant activity is another prime consideration for mimetic design. Furthermore, heparin mimetics are designed to tackle the heterogeneity of heparin, the rational being homogeneous structures could lead to enhanced potency and greater specificity. The ultimate goal in designing heparin mimetics is the removal of unwanted biological activities and to maximize their therapeutic benefits for the disease being targeted.

Major advances in the field of carbohydrate synthesis such as building block preparation (one-pot multi-step procedures), coupling reactions, and the development of convergent strategies for coupling key building blocks, has led to an increase in the synthesis of complex oligosaccharides which can act as heparin mimetics [16,17,18,19,20]. Such synthetic methods allow the preparation of tailor-made saccharides, with customized sizes and functional groups. These tailor-made saccharides can be used for SAR studies on a specific biological target. A number of discovery programs, including our own, have used this approach for hit optimization of heparin/heparan sulfate mimetics for various therapeutic uses such as anticoagulation, angiogenesis modulation, anti-cancer metastasis, and anti-inflammation.

This review will highlight the advances in the development of heparin mimetics for various clinical purposes, with a focus on the potential of using these compounds as drugs.

## 2. Structure and Diversity of Heparin Mimetics

There is great diversity in the structure and biological activities of the heparin mimetics that have been reported in the literature. Most of the mimetics created to date are carbohydrate based mimetics, although non-carbohydrate mimetics have also been reported and there is a growing number of mimetics that are a combination of carbohydrates and aglycones. The anionic charge of heparin mimetics has generally been introduced by sulfation and rarely by phosphorylation or carboxylation. The main classes of heparin mimetics that have been reported in the literature are: (a) modified polysaccharides; (b) synthetically sulfated oligosaccharides; (c) oligosaccharide-aglycone conjugates; and (d) non-carbohydrate-based sulfated mimetics. For a detailed description of these classes the reader is directed to the review by Coombe and Kett [21].

When developing mimetics that are directed towards particular therapeutic needs certain structural features are a necessity for modulating biological function. Some such structural features include:

- Size/molecular weight: The first step in any synthetic strategy for a heparin mimetic directed towards a specific target is to determine the size of the structure that is most likely to have the required biological activity. This decision requires knowledge of the shape and size of the heparin binding site on the protein target. Not all heparin binding sites resemble the small pockets on proteins surfaces that are traditionally targeted by drugs; for example, they may be a face on a protein surface. If the latter is true, the size of the heparin-like structure that is required for binding, and for modulating the protein’s function, will be larger than a disaccharide or a tetrasaccharide. It can also be that larger structures are required to provide the correct orientation of the entity that actively engages with the protein. Hence, smaller analogues may not always produce the anticipated increase in selectivity and potency.
- Heterogeneity: Heterogeneous mixtures are more likely to display a broad range of biological activities than structurally similar homogeneous products, but the technical challenges associated with their reproducible synthesis and the characterization of heterogeneous mixtures are far greater. This means proof of reproducible synthesis is required once a heterogeneous mimetic enters clinical development. Accordingly, current trends are towards the synthesis of structurally homogeneous heparin mimetics.
- Pattern and extent of sulfation: It is well known that the extent of sulfation influences the strength with which heparin or HS fragments bind proteins. This was concluded from studies where HS fragments were eluted off protein affinity columns with varying salt concentrations; the highly sulfated structures eluting at higher salt concentrations [22]. These studies also showed that when HS fragments bound some proteins, like Fibroblast growth factor-1 (FGF-1), a higher degree of sulfation did not necessarily translate into the fragments displaying higher affinity binding. Thus, not only the number of sulfates but also the positions of the sulfates were important [22]. We have also shown that the pattern and extent of sulfation has a marked effect on the location on a protein where heparin fragments prefer to bind and that not all fragments that bind affect the protein’s activity in the same way [15]. Given these findings with heparin fragments, it is probable that heparin mimetics will similarly vary in their activities in accordance with the patterns of sulfation. Techniques to control the degree of sulfation include; the choice of starting material, selective sulfation and limiting reaction conditions. Similarly, careful selection of different carbohydrate starting materials can result in different patterns of sulfation in a mimetic.
- Linkage patterns: The influence of anomers, or of linkage patterns on the biological activity of a polysaccharide can also be explored by careful selection of the starting material. Both of these aspects of GAG structure contribute profoundly to their solution structures, and in all probability also to the structures GAG fragments adopt when bound to proteins. The torsion angle values are altered by glycosidic linkages and the anomeric configuration of the linkage, and even small differences in these angles can contribute to differences of the structure in solution. This is illustrated by the more bent solution structure of HS compared to that of heparin [23], although here sulfation and monosaccharide differences also contribute.
- Flexibility of the backbone: Polysaccharide chains are relatively inflexible due to the limited rotations allowed about a glycosidic linkage. Thus, more flexible heparin mimetics are synthesized by chemical modifications such as glycol splitting. Furthermore, synthetic non-carbohydrate chemical linkers of varying degrees of flexibility can be employed to link short carbohydrate chains, resulting in more flexible heparin mimetics. This approach was used to produce HS-mimics that bound interferon-γ. Two highly sulfated octasaccharide HS fragments linked by a spacer of 10 polyethylene glycol repeats were found to efficiently bind interferon-γ [24]. It was argued that when linked, the sulfated regions acted in a concerted manner and formed a functional unit, whereas when unlinked the octasaccharides did not bind efficiently.

Heparin mimetics are a group of compounds that have been created to combat the promiscuity and polypharmacy of heparin. Possible strategies that can be used to address this problem include; increasing the potency of a particular mimetic such that the dosage can be decreased to a point where harmful side effects are insignificant. Alternatively, increasing binding selectivity could eliminate undesired protein interactions of a mimetic.

## 3. Heparin Mimetics as Anticoagulants

**Figure 2:**
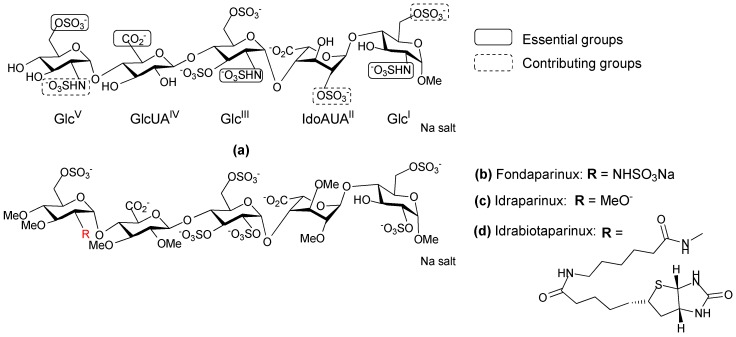
Chemical structure of heparin pentasccharide derivatives. (**a**) The antithrombin III binding pentasccharide motif of heparin; (**b**–**d**) Structure of synthetic analogues of the antithrombin III binding site of heparin.

Heparin in its unfractionated form has been used as an anticoagulant drug for over 80 years. SAR studies have shown that a unique pentasaccharide sequence, termed the “antithrombin III-binding domain”, is primarily responsible for the anticoagulant activity of heparin. The antithrombin III-binding domain contains a very specific pattern of negatively charged groups (*O*-sulfonates and carboxylates) surrounded in standard heparin by a repetitive sequence composed of 2-*O*-sulfated IdoA linked to a 6-*O* sulfated glucosamine (Figure 2a). Although heparin is highly effective and inexpensive, it has several undesirable qualities as a therapeutic. Firstly, it is a heterogeneous mixture of compounds extracted from porcine or bovine mucosa, and it carries a potential risk of contamination as is illustrated by the incident which occurred in 2007–2008 [25,26]. Secondly, heparin chains vary in size, anticoagulant activity, and in their ability to bind various plasma proteins; as a consequence heparin displays a variable dose-response relationship amongst patients and requires active monitoring to fine-tune the dosage [27]. Third, approximately 3% of patients undergoing prolonged heparin therapy experience severe autoimmune responses [28]. These limitations have led to the development of a variety of low molecular weight heparin like anticoagulants with more homogenous composition and predictable pharmacokinetic properties.

Anticoagulants based on heparin are the drugs of choice in the therapy and prophylaxis of thromboembolic diseases. The anticoagulant market for heparin mimetics has been very active over the past few decades due to the development of new compounds. Several reviews have been published describing the SAR and mechanism of action of these heparin mimetic anticoagulants [29,30,31].

In 2001, GlaxoSmithKline (GSK; Brentford, UK) registered Fondaparinux as a new antithrombin III drug under the name Arixtra [32]. It is the methyl glycoside analogue of the natural antithrombin III binding pentasaccharide in which the acetamido is replaced by a sulfoamino group on the GlcV unit (Figure 2b). The specificity and the binding strength of Fondaparinux to antithrombin III, can be attributed to the presence of the methyl groups which prevent non-specific binding to plasma proteins. Fondaparinux has a linear pharmacokinetic profile and a longer half-life, compared to LHWHs. In addition, it does not induce immune thrombocytopenia. Fondaparinux is now approved for the prophylaxis and treatment of venous thromboembolism (VTE) in virtually all Western countries, and is increasingly being used as a substitute for LMWHs. In the search for antithrombotic carbohydrates with reduced synthetic complexity and tailor-made pharmacological properties, attention was directed to a novel class of ‘non-glycosaminoglycan’ analogues. Initially the synthesis of Fondaparinux was performed in about 50 chemical steps [33], and other synthetic methods have been explored to obtain a straightforward synthetic sequence with fewer steps that could also be used to obtain analogues. Indraparinux (Figure 2c) is a synthetic pentasccharide analogue of Fondaparinux, in which the hydroxyl groups are methylated and the *N*-sulfate groups are replaced by *O*-sulfates [34]. It can be prepared from glucose in approximately 25 synthetic steps in a highly convergent manner. The strategy used for the synthesis of Idraparinux consists of assembling the Glc^V^ on a suitably protected GlcUA^IV^-Glc^III^-IdoUA^II^-Glc^I^ tetrasaccharide [35]. Idraparinux was not only synthesized using fewer synthetic steps but it also gave rise to a new class of antithrombotic agent that specifically inhibited Factor Xa and lacked activity against thrombin. It was developed for the treatment and secondary prevention of VTE, as well as for the prevention of thromboembolic events associated with atrial fibrillation. Some of the clinical advantages of Idrapainux include: (1) A linear pharmacokinetic profile and a longer half-life than Fondaparinux; (2) It has consistent effects as it is not metabolized and is completely bioavailable; (3) It does not bind to plasma proteins and in particular does not bind platelet factor 4 (PF4), which makes the development of immune thrombocytopenia extremely unlikely [36]. However, Idrapainux has no neutralizing agent thus its antithrombotic activity cannot be reversed, unlike heparin. This can lead to severe complications in some cases.

Idrabiotaparinux (Figure 2d) is a novel, long-acting, synthetic anticoagulant, which has a similar chemical structure to Idrapainux but with an added biotin segment. The biotin segment of Idrabiotaparinux enables the neutralization of the drug using avidin as a neutralizing agent. Avidin is a tetrameric protein derived from the egg white of many species. Injection of avidin can trigger the immediate elimination of biotinylated Idrabiotapariux from the blood stream of humans and animals, resulting in neutralization of the antithrombic activity [37,38]. However, a systematic review reported a lack of sufficient evidence to clarify whether Idraparinux and Idrabiotaparinux are as effective and safe as the standard warfarin treatment for VTE [39]. As a result, Sanofi-Aventis (Paris, France, now known as Sanofi) halted the development of Idrabiotaparinux for arterial fibrillation at the phase III clinical trial stage.

**Figure 3:**
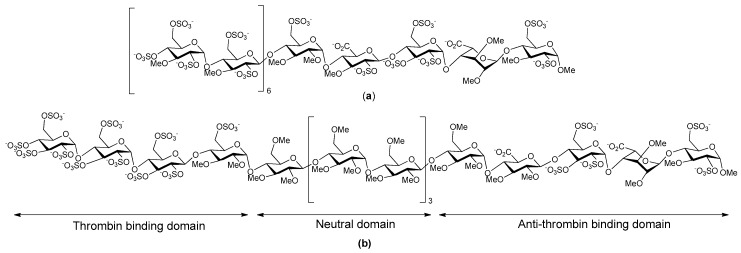
Chemical structure of (**a**) 17-mer saccharide; (**b**) SR123781.

A number of years ago now Petitou et al. synthesized a family of heparin mimetic oligosaccharides that were able to inhibit thrombin as well as bind antithrombin III, the aim being to obtain more potent, well-tolerated antithrombic drugs [40]. Unfortunately, like thrombin inhibition, undesirable interactions with plasma proteins are directly correlated with the charge and the size of the oligosaccharide. So the compounds were designed to discriminate between thrombin and other proteins, particularly PF4. A multistep converging synthesis was used to obtain sulfated oligosaccharides that met these requirements. Petitou et al. reasoned that thrombin inhibition should be obtained when the saccharide was long enough to accommodate antithrombin III and thrombin at the same time. Accordingly, they synthesized a “non-GAG” series in which *N*-sulfate groups were replaced by *O*-sulfate groups, and hydroxyls were alkylated. These structural modifications fully preserved the specific binding to antithrombin III yet they drastically simplified the synthesis. This work led to the synthesis of a 17-mer oligosaccharide (Figure 3a) which was 5- to 10-fold more potent than standard heparin and LMWHs in models of both venous and arterial thrombosis. The design of this oligosaccharide was based on the following considerations: (1) a pentasaccharide sequence is required to bind and activate antithrombin III towards Factor Xa (FXa) and thrombin inhibition; (2) the separate thrombin binding-domain must be two to three disaccharides long; (3) a chain length of 17 saccharide units is required for notable thrombin inhibition meaning the two binding domains could be separated by a spacer; and (4) as the six or eight units of the central saccharide spacer were not critically involved in interacting with either antithrombin III or with thrombin, the charge on these units could be suppressed without affecting the anticoagulant activity. The 17-mer saccharide that resulted displayed a very simple elimination profile compared to heparin and LMWHs, owing to the limited number of proteins with which it interacted. The publication describing this 17-mer saccharide attracted a lot of excitement, and at the time it was thought such longer oligosaccharides had the potential to address some of side effects of other anticoagulant drugs that were on the market [40,41].

SR123781 is a short acting synthetic hexadecasaccharide, developed from the work of Petitou et al. [40]. It is an indirect antithrombin III dependent inhibitor of FXa (Figure 3b). SR123781 is an analogue of heparin and was obtained from glucose through a convergent synthesis; it comprises an antithrombin binding pentasaccharide, a thrombin binding sulfated tetrasaccharide, and a neutral methylated hexasaccharide linker sequence, with *N*-sulfated groups replaced by *O*-sulfates, and alkylated hydroxyl groups in the antithrombin III binding domain. SR123781 was found to be more potent than heparin and Fondaparinux in a number of different animal models for arterial venous thrombosis with a high affinity for human antithrombin [42]. Furthermore, SR123781 displayed prolonged anti-FXa and antithrombin activity after intravenous and subcutaneous administration to rats, rabbits and baboons. It also inhibited thrombus formation in experimental in vivo models and had a favorable antithrombotic:bleeding ratio compared to heparin [43]. Even though SR123781 progressed to phase IIb clinical trials, its development was discontinued following the success of the heparin mimetic AV5026.

AV5026 (Semuloparin, Sanofi-Aventis) is a complex mixture of oligomeric ultra-low-molecular-weight heparin fragments (molecular weight 2000–3000 Da) with a polydispersity index of approximately 1.0. It is synthesized by partial and controlled chemoselective depolymerization of porcine unfractionated heparin. It has a novel antithrombotic profile resulting from high anti-FXa activity and residual anti-Factor IIa (FIIa or thrombin) activity in comparison to heparin and LMWH [44]. This unique physiochemical profile of AV5026 is the result of a highly selective depolymerization of heparin by a phosphazene base. AV5026 demonstrated a dose-dependent antithrombic effect with similar activity compared to the marketed LMWH, enoxaparin, in well-established thrombosis models in rats, rabbits and dogs [45]. Clinical evaluation of AV5026 in patients undergoing orthopedic surgery (TREK-study) demonstrated a significant dose response for the prevention of venous thromboembolism [46]. Although, clinical data suggested that AV5026 could represent a new alternative for the prevention of thrombosis with an improved benefit-risk profile compared with classic heparinoids and LMWHs, its development was stopped in 2012.

**Figure 4:**
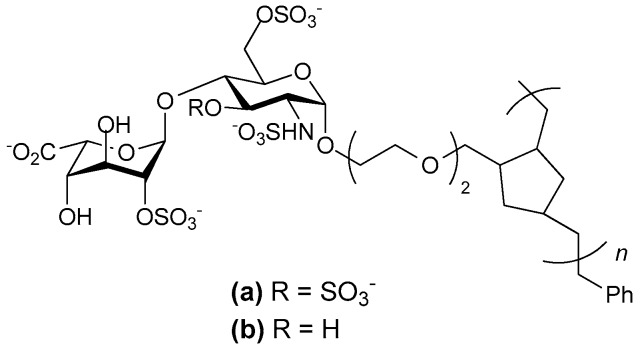
Chemical structure of tailored glycopolymers as anticoagulant heparin mimetics.

Several decades of intensive research has led to the discovery of several synthetic heparin mimetics with anticoagulant activity. Structure based approaches gave insights into the mechanism of heparin-induced activation of antithrombin III and identification of the pentasaccharide sequence in heparin that bind antithrombin III with high affinity. This then led to the discovery of LMWH or fragments of heparin with higher potency and a longer half-life with fewer side effects. However, the search for novel heparin mimetics with increased affinity have proven to be a formidable challenge and constructing simplified analogues of the unique heparin binding pentasccharide is a complicated endeavor. Recently the notion of synthesizing glycopolymers with hydrocarbon backbones that carry pendant well defined heparin disaccharides was explored. The aim was to achieve efficient synthesis of defined heparin-like compounds that retained the anticoagulation activity of intact heparin. Hsieh-Wilson and her colleagues synthesized a compound that surpassed the anticoagulant activity of heparin (Figure 4) [47], showing greater anti-Factor Xa and 100-fold greater anti-FIIa activity. This compound comprised tetrasulfated disaccharides consisting of 2-*O*-sulfated l-iduronic acid and glucosamine with 3-*O* and 6-*O*-sulfation as well as *N*-sulfation. The sulfation reactions were performed prior to polymerization and the polymerization was controlled to produce a glycopolymer of 45 repeats. This compound also strongly bound PF4, a result in accordance with other reports indicating that long negatively charged species that are not glycosaminoglycans may bind this chemokine [48,49]. When the polymerization was restricted to 30 repeats anti-FXa and anti-FIIa activities were markedly lower than that of both heparin and the longer polymer. Moreover, loss of the 3-*O*-sulfate from the glucosamine abrogated the anti-FXa activity [47], pointing to the importance of this sulfation pattern for antithrombin III binding. It is particularly interesting that a collection of disaccharides carrying this motif arranged in a pendant fashion on a polymeric scaffold are sufficient to bind antithrombin III and trigger anti-FXa activity, as previous studies with heparin have indicated that a pentasaccharide structure containing a 3-*O*-sulfated glucosamine was required [40]. Nevertheless, it remains to be seen as to the clinical usefulness of this anticoagulant heparin mimicking glycopolymer.

## 4. Heparin Mimetics in Cancer

HS are present on the surface of all eukaryotic cells, including tumor cells and the stromal cells surrounding a tumor, which are important for tumor survival. The HS on tumor cell surfaces have been shown to be vital for many aspects of tumor phenotype and tumor development, including cell transformation, tumor growth, cell invasion and metastasis. Both clinical and animal model studies indicate that heparin mediates anti-tumor activity [50,51,52,53]. Furthermore, a number of retrospective and prospective clinical studies have shown that heparin therapy may prolong the survival of cancer patients across a variety of solid tumor types [54,55]. However, exploitation of heparin’s anti-tumor activities are limited by its anticoagulant activity. Therefore, research has been directed at developing heparin mimetics with limited anticoagulant activity but with the retention of heparin’s anti-tumor activity.

Heparanase is an endoglycosidase which cleaves the HS side chains of heparan sulfate proteoglycans (HSPG) in the ECM surrounding tumor cells. The degradation of ECM facilitates the spread of tumor cells by enabling them to enter into, and escape from, blood vessels and lymphatics. In addition, heparanase is known to exhibit pro-angiogenic properties, i.e., stimulate the growth of new blood vessels from pre-existing blood vessels that surround tumors [56]. Thus, drugs targeting heparanase have been under investigation by both academic and industry based laboratories. As a result, numerous sulfated sugar molecules including heparin mimetics have been identified as selective inhibitors of heparanase [57]. In addition to heparanase, fibroblast growth factors (FGFs) and vascular endothelial growth factor (VEGF) are essential mediators of angiogenesis and thus are also attractive targets for drug discovery [58]. These growth factors are sequestered to the extracellular matrix by binding to HS in the matrix associated HSPGs, and they are released by heparanase. FGFs and VEGF initiate the cell signaling cascades that lead to angiogenesis by forming ternary complexes with HS and their particular cognate cell surface receptors. Thus, inhibiting angiogenesis by targeting the HS and heparin binding sites on these growth factors with heparin mimetics is considered to be a viable therapeutic strategy for cancer [59,60,61].

**Figure 5:**
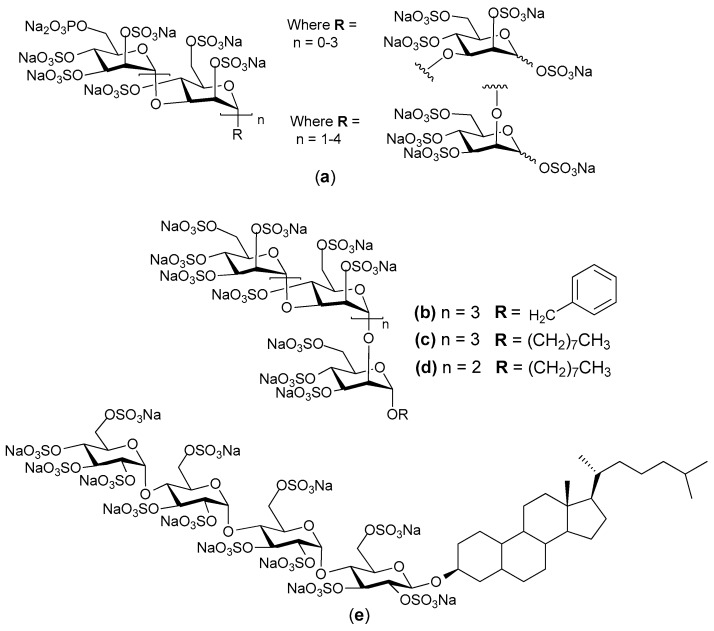
(**a**) Generalized chemical structure of PI-88; (**b**–**d**) Analogues of PI-88; (**e**) Chemical structure of PG545.

PI-88 (Progen Pharmaceuticals Ltd. (Brisbane, Australia) is one such inhibitor. It is the product of exhaustive sulfation of the oligosaccharide phosphate fraction of the extracellular phosphomannan (derived from the yeast *Pichia (Hansenula) holstii* NRRL Y-2448) [62,63]. It is a heterogeneous material being primarily composed of sulfated phosphomannopentaose and phosphomannotetraose oligosaccharides carrying variously 10–13 sulfates [14,64]. A detailed analysis of the non-sulfated starting material from which PI-88 was prepared has recently been published [65]. PI-88 exerts its antimetastatic effects by inhibiting heparanase and so the cleavage of HS in the ECM and the release of angiogenic growth factors. PI-88 also binds competitively to growth factors, such as FGF-1 and FGF-2 and VEGF to exert an anti-angiogenetic effect [66]. PI-88 has been tested in phase II clinical trials for liver cancer and has shown efficacy as an adjuvant for postsurgical hepatocellular carcinoma (HCC) [67,68]. It is currently in phase III HCC clinical studies, but has not yet been approved for routine clinical use. Phase I and Phase III clinical trials of PI-88 in patients with advanced melanoma also demonstrated noteworthy activity [69]. PI-88 is generally well tolerated, but is does have the common toxicity issues of thrombocytopenia and thrombosis, injection site hemorrhage and other bleeding events. Several analogues of PI-88 have been synthesized with the aim of altering the pharmacokinetic properties in a favorable manner to result in less frequent dosing whilst maintaining biological activity. As such, the analogues were based on a single pentasaccharide backbone for ease of synthesis and evaluation of biological activity. The compounds in the initial series of analogues were mostly glycosides of the major pentasaccharide found in a (Figure 5) [70,71]. Compounds such as **b c**, and **d** (Figure 5) exhibited biological activity similar to that of **a**, but with improved pharmacokinetics in a rat model. Furthermore, both compounds **a** and **b** were shown to be potent inhibitors of in vivo angiogenesis in two separate murine models. Additionally, the introduction of lipophilic modifications resulted in significant attenuation of anticoagulant activity, a common side effect of heparin/HS mimetics including PI-88. This data supports the continued development of heparin/HS mimetics of this type as antiangiogenic, anti-cancer agents.

Progen Pharmaceuticals Ltd. then designed a series of compounds named PG500, which are second-generation versions of PI-88. The PG500 series of compounds are anomerically pure, fully sulfated and are single entity oligosaccharides attached to a lipophilic moiety, e.g., aglycone, at the reducing end of the molecule [72]. Some of the compounds from this series are more potent inhibitors of angiogenesis and metastasis than PI-88 and have shown strong anti-tumor activity in some aggressive tumor models [73]. These compounds are believed to interfere with angiogenesis via inhibition of VEGF and FGFs, and with metastasis via inhibition of heparanase. The lead molecule of this series is PG545, which was selected based on its efficacy, pharmacokinetics, toxicology and ease of manufacture [74].

PG454 is a synthetic, fully sulfated tetrasaccharide functionalized with a cholestanyl aglycone (Figure 5e) [75]. It is currently in phase I clinical trials and has shown potent inhibition of heparanase with low anti-coagulant properties. Interestingly, the cholestanyl group increased the affinity of PG545 for heparanase over that of a non-functionalized derivative. It appears this hydrophobic group allows PG545 to bind to a hydrophobic pocket near to the active site of the enzyme in addition to binding to the basic amino acids that are involved in HS binding [76]. PG545 has been shown to exhibit a long plasma half-life and shows activity in multiple murine models of the cancers of breast, prostate, liver, lung and colon, as well as head and neck cancers and melanoma [75]. Recent publications have shown that PG545 has activities in addition to those attributed to its effects on heparanase and the growth factors VEGF and FGF-2. For example, the anti-lymphoma effect of PG545 in vivo seems to require NK (natural killer) cell activation and this activation involves PG545 acting via TLR9 (toll-like receptor-9) to trigger dendritic cells to release IL-12, which is necessary for PG545 activation of NK cells [77]. In addition, PG545 was shown to act on the Wnt pathway in a pancreatic ductal adenocarcinoma model. PG545 bound directly to Wnt3a and Wnt7a, thereby blocking their interactions with their receptors. This inhibition of Wnt signaling within tumor cells led to reduced levels of β-catenin, which caused a reduction in the expression of VEGF, matrix metalloproteinase (MMP)-7 and Cyclin D1, as well as triggering apoptosis of the tumor cells [78]. The multiple activities of PG545 mirror the ability of heparin to also act on multiple pathways and points to the likely polypharmacy of most heparin mimetics.

**Figure 6:**
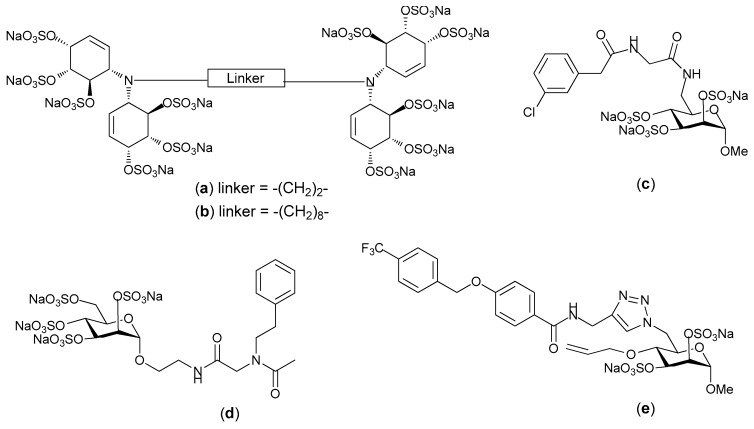
Small molecule heparin/heparan sulfate (HS) mimetics as potential inhibitors of fibroblast growth factors (FGF)-and/or vascular endothelial growth factor (VEGF) mediated angiogenesis for the development of novel cancer therapeutics; Structure of potent (**a**,**b**) linked cyclitol; (**c**,**d**) compounds from Ugi library; (**e**) compounds synthesized via click chemistry.

Heparin/HS binding proteins interact with particular preferred structural motifs within heparin and HS, and the extent to which a particular motif is a requirement for binding varies from one heparin binding protein to another. Some heparin binding proteins are very specific in their requirements (e.g., antithrombin III), whereas others are less specific (e.g., compare FGF-1 and FGF-2 [79]); thus heparin/HS mimetics are often designed with a view of selectively blocking protein-GAG interactions. To explore this, Parish and co-workers synthesized a homogenous structurally well define HS mimetic family of sulfated linked cyclitols. They then compared the ability of 15 different sulfated linked cyclitols to bind to 10 functionally diverse proteins [80]. This relatively simple panel of mimetics provided considerable information regarding the patterns and orientations of anionic groups that are recognized by different proteins. Specifically, it demonstrated that spatial separation of anionic groups within the HS mimetics plays a critical role in determining the specificity of interactions. For example, compounds **a** and **b** (Figure 6), which only differ by the length of their alkyl chain spacer exhibited different inhibitory activity towards growth factors. Compound **a** was found to be strong inhibitor of FGF-1, with modest inhibition of FGF-2. Whereas compound **b** was a strong inhibitor of FGF-2 and VEGF with modest inhibition of FGF-1. Ferro and co-workers extended this work by synthesizing a library of mimetics with simple ionic binding motifs on a monosaccharide scaffold based on d-mannopyranose that could “anchor” the ligand to the HS-binding site on the targeted protein [81]. This library of mimetics was synthesized by harnessing the powerful Ugi four component reaction for decorating the scaffold with linkers containing a diverse range of functional groups. The affinities of these monosaccharide mimetics for angiogenic HS-binding growth factors were close to those generally observed for polysulfated di-or tetrasccharides. From this study a clear trend for proteins to prefer particular structures was evident, for example the presence of an aromatic group was favored by FGF-1 and VEGF. This supported the previous observation that lipophilic modifications can improve affinity for VEGF and FGF-1 but not FGF-2 [82].

Ferro and co-workers also synthesized an 18-membered library of small molecule HS mimetics via click chemistry. These mimetics were targeted against the angiogenic growth factors FGF-1, FGF-2 and VEGF [83]. The library of mimetics was designed with the specific aims of firstly, identifying the critical sulfate groups for binding on the monosaccharide ring, with the view to decreasing the overall sulfation without loss of activity, and secondly, to enable the exploration of possible non-ionic binding surfaces around the primary binding site on the proteins; the primary binding site being comprised of clusters of basic, positively charged amino acids. 6-azido-6-deoxy-α-d-mannopyranoside was selected as the building block for the synthesis of this library of heparin mimetics. The library was made more diverse by incorporating various alkyne derivatives into functional motifs and by employing a Swern oxidation-Witting olefination sequence to synthesize functional motifs (e.g., aromatic groups) with varied geometrical representation to produce different isomers. Collectively this diversity allowed exploration of the size and position of hydrophobic or hydrogen bonding regions near to the basic amino acids which comprised the primary binding site. The binding studies indicated that affinity and structural specificity of the mimetic was increased by the incorporation of non-anionic motifs. A lead compound was identified from the library with micromolar binding affinity towards FGF-1 and VEGF and good selectivity over FGF-2 [83]. This work illustrates the power of combining an anionic sugar structure with an aromatic spacer, of an appropriate length and rigidity, to position a polar group at a site on the protein removed from the binding hot-spot that recognizes the sulfate groups, for enhancing the specificity of the heparin mimetic. Interestingly, this strategy provided a rapid method to probe the chemical space around a binding hot-spot, which could then be used to differentiate a family of similar proteins by their mimetic binding capability.

Recently, “small glycol” drugs which are heparin mimetics have been identified as exhibiting anti-cancer properties. These molecules are small, synthetic oligosaccharides with potent affinity and selective inhibition of several growth factors and proteins involved in tumor growth and propagation. As such, a library of more than 100 synthetic oligosaccharides of different sizes containing various substitutions has been evaluated for their specificity and efficacy in inhibiting cell proliferation and migration and in vitro endothelial tubule formation. EP80061 is the lead compound in this series which has shown potent anti-metastatic effects in a disseminated tumor model in C571B1/6 mice [84]. The structure of this series is yet to be disclosed.

**Figure 7:**
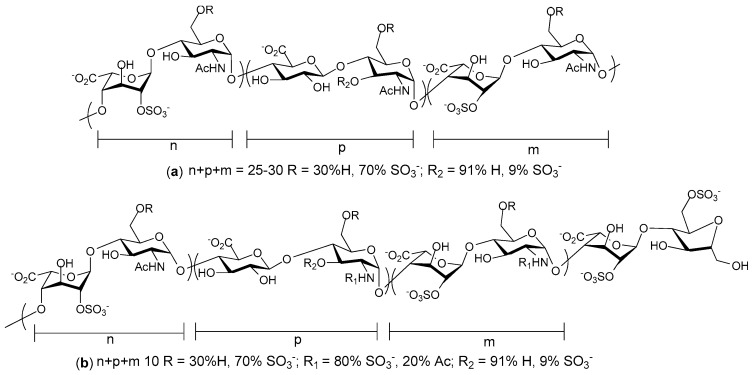
Schematic representation of (**a**) SST0001 (roneoparstat) (**b**) M402 (necuparanib). The actual structures may retain the microheterogeneity of the original heparin and low-molecular-weight-heparin (LMWH) [89].

Sigma-Tau Pharmaceuticals (Gaithersburg, MD, USA) developed a heparin mimetic, SST0001 (Roneparstat, Figure 7a) which is currently in phase I clinical studies for advanced multiple myleoma. SST001 is an *N*-acetylated, glycol-split high molecular weight heparin that also exhibits low anticoagulant activity and selective inhibition of heparanase [85]. It is obtained from standard porcine mucosal heparin after total *N*-desulfation, *N*-acetylation, controlled periodate oxidation, and finally borohydride reduction (the sequence of the last two steps is called glycol-splitting). In preclinical studies, SST0001 showed a significant anti-myeloma effect in in vivo models of multiple myeloma [86]. The combination of SST0001 with irinotecan, a cytotoxic agent, exhibited potent activity against sarcoma xenografts with all animals showing marked tumour regression and a number of animals had no apparent disease at the completion of the experiment, whereas either drug alone only delayed tumour growth [87]. The latter study demonstrated that in vivo SST0001 administration caused reduced phosphorylation of a number of receptor tyrosine kinases (EGFR, ERBB4, INSR and IGF1R), providing evidence that this heparin mimetic may influence intracellular signaling as well as block heparanase.

Momenta pharmaceuticals presented anti-metastatic preclinical data for a heparin mimetic, M402 (Necuparanib, Figure 7b). M402 is a low molecular weight heparin, resulting from depolymerization of heparin and further oxidation and borohydride reduction. In vitro and in vivo studies of M402 showed reduced anticoagulant activity and inhibition of tumor metastasis through the modulation of factors, such as P-selectin, VEGF, and FGFs [88]. M402 has shown statistically significant survival benefits in animal models when used as a monotherapy or in a combination with other chemotherapeutics. Furthermore, mice treated with M402 showed reduced epithelial-to-mesenchymal transition, a key step in the progression of tumor cells towards a more invasive phenotype. Currently, M402 is in phase II clinical trial for pancreatic cancer.

**Figure 8:**
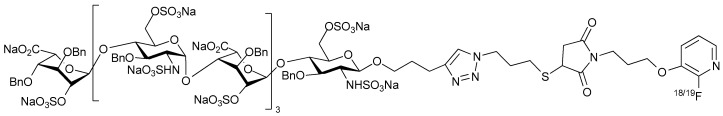
Chemical structure of the conjugated and radiolabeled octasaccharide-based HS mimetic.

Endotis Pharma (Romainville, France) reported the synthesis of an octasaccharide-based heparin mimetic capable of antagonising angiogenic proteins that are known to be involved in cancer progression and angiogenesis associated with tumor growth [90,91]. The mimetic is expected to interfere with two major processes in tumor progression: (1) angiogenesis, in part mediated by growth factors, or endothelial progenitor cell recruitment; and (2) metastasis mediated by heparanase activity. The octasaccharide was assembled from three different disaccharide units which were synthesized from three different monosaccharides in sufficient quantities and with appropriate protecting groups. A fluoropyridinilated derivative of the sulfated octasaccharide heparin mimetic was then prepared by linking a triazole conjugated with FPyME to the reducing terminus (Figure 8). The use of [^18^F]FPyMe allowed the incorporation of radiolabeled fluorine-18 which allows in vivo evaluation of the compound and is extremely useful for performing the ADMET assays required for drug development. Pharmacodistribution studies revealed that following injection into rats this heparin mimetic accumulated in the kidneys and to a lesser extent the liver and at later time points the bladder, indicating likely urinary excretion. Radioactivity in other organs was low but clearance from the blood vascular system was slow (t_1/2_ was greater than 90 min). The in vivo properties of this mimetic are appropriate for an anti-cancer drug.

Heparin mimetics designed for specific targets are expected to act preferentially on specific proteins such as growth factors of the FGF family and VEGF, and heparanase, which are overexpressed in cancer, but as was seen with PG545 other activities may well be detected for some of these compounds. The design of precisely tailored heparin mimetics is likely to require both anionic and non-anionic structures, the latter structures binding to the protein target outside of the basic region that is recognized by heparin and other GAGs.

## 5. Heparin Mimetics as Anti-Inflammatories

It has long being recognized that heparin has range of biological effects in addition to its well characterized anticoagulant properties, and possibly one of its best known non-anticoagulant activities are its anti-inflammatory effects [92,93,94]. The anti-inflammatory properties of heparin and heparin mimetics have primarily, but not exclusively, been linked to their ability to interact with three different types of proteins: complement system proteins, selectins and chemokines; each of which function in different ways to facilitate inflammation [95,96,97]. The potential of heparin as an anti-inflammatory drug is supported by a number of small, and several modestly sized clinical trials. Heparin has been shown to benefit patients with arthritis [98], inflammatory bowel disease [99,100], allergic rhinitis [101] and bronchial asthma [102,103]. However, the use of heparin as an anti-inflammatory agent has been hindered by its anticoagulant proprieties. Since the anti-inflammatory potential of heparin is independent of its anticoagulant properties, the development of novel non-anticoagulant heparin mimetics but with the anti-inflammatory properties of heparin is an attractive possibility. Thus, considerable effort has been directed towards the development of heparin mimetics with the potential to act as anti-inflammatory agents.

**Figure 9:**
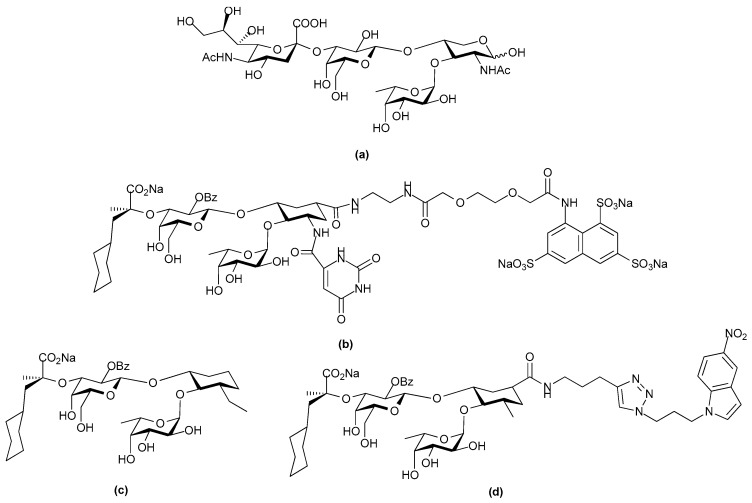
Chemical structure of (**a**) Sialyl Lewis^x^ (sLe^x^); (**b**) GM-1070, sLe^x^ mimic in clinical trials for treatment of vaso-occlusive crisis in sickle cell disease patients; (**c**) sLe^x^ mimic that maximizes conformational pre-organization of the binding determinants; (**d**) sLe^x^ mimic designed using fragment based discovery techniques with improved binding kinetics.

It is well known that selectins play a key role in the early stages of an inflammatory response [104], and so antagonists of selectins have the potential for being valuable therapeutic agents for various inflammatory diseases. The Sialyl Lewis^x^ (sLe^x^) motif provides a lead structure for the search of E-selectin antagonists since it is a component of all the physiological receptors of the selectins. This motif, NeuAcα2-3Galβ1-4(Fucα1-3)GlcNAc, on its own is not a selectin receptor, rather additional structures are also required for binding. For example, P-selectin binds sLe^x^ on a threonine residue and an adjacent sulfated tyrosine on its ligand, P-selectin glycoprotein ligand-1; L-selectin binds O-linked SLe^x^ with a sulfate on the 6-hydroxyl of the SLe^x^ GlcNAc, and E-selectin binds glycosphingolipids carrying the terminal glycan structure: NeuAcα2-3Galβ1-4GlcNAcβ1-3 [Galβ1-4Fucα1-3)GlcNAcβ1-3]_2_-R [105]. GMI-1070 (Rivipansel, developed by GlycoMimetics Inc., Rockville, MD, USA. Figure 9b) is a novel small molecule sLe^x^ mimetic pan-selectin antagonist [106]. GMI-1070 was developed by rational design, focusing on the conformation of sLe^x^ when it is bound to E-selectin, as determined by nuclear magnetic resonance. In GMI-1070 the oligosaccharide fragments of sLe^x^ were replaced with a conformationally restricted linker which reproduced the three-dimensional (3D) features of sLe^x^. GMI-1070 contains a cyclohexane core structure with a carbohydrate branching motif linked to a benzyl amino sulfonic acid residue [107]. The extended sulfated domain was designed to allow binding of P- and L-selectins as well as E-selectin. Although it binds all three selectins its ability to block the binding of the natural selectin ligands, sLe^a^ and sLe^x^ to immobilized selectins was far greater with E-selectin than with L-selectin or P-selectin, being approximately 10-fold more sensitive with an IC_50_ of 4.3 µM. Selectins facilitate leukocyte rolling on endothelial cells of the vasculature, which is the first step in leukocyte migration from the blood and into the tissues in an inflammatory response. The administration of GMI-1070 into mice with sickle cell disease caused an increase in leukocyte rolling, which is a characteristic of E-selectin inhibition. Moreover, GMI-1070 reduced the number of leukocytes adhering to the venular endothelium and also inhibited red blood cell-leukocyte interactions and vascular occlusion. It is currently undergoing clinical trials for treatment of vaso-occlusive crisis in people with sickle cell disease. It has been demonstrated to have a good safety profile, a serum half-life of 7–8 h and greater than 90% of the drug is excreted intact [106,108]. Phase II studies revealed the drug reduced the time to resolution of vaso-occlusive events (not considerably significant) and improvements were also observed in other aspects, like time to hospital discharge [109]. Phase III trials have commenced for this drug.

Recently, Ernst and co-workers dissected the role of the cyclohexane core structure of the natural SLe^x^ ligand and correlated (by nuclear magnetic resonance spectroscopy (NMR) and molecular dynamic calculations) the affinities with which it binds E-selectin with the flexibility and the degree of pre-organization of the pharmacophores in the bioactive conformation [110]. In SLe^x^, the hydroxyl groups of the fucose moiety, the 4- and the 6-hydroxyl of the galactose moiety and carboxylic acid group of the sialic acid residue act as pharmacophores. Whereas the *N*-acetylglucosamine (GlcNAc) moiety serves as 3D spacer to position L-fucose underneath the β-face of d-galactose. This 3D orientation and pre-organization of the pharmacophores are extremely important for high affinity binding [111]. Ernst and co-workers used a molecular design strategy which involves introducing conformational restriction which then limits the degrees of freedom a molecule can lose upon binding, to synthesize a library of high-affinity E-selectin antagonists. These antagonists were synthesized with a cyclohexane linker which acts as GlcNAc mimic of the native SLe^x^ ligand. Using Surface Plasmon Resonance (SPR) and Saturation Transfer Difference NMR experiments, they showed that addition of hydrophobic substituents on the cyclohexane linker improved the affinity by forcing the adjacent fucose moiety into the bioactive conformation. In this manner, the affinity of the native SLe^x^ (Figure 9a) was improved more than 660-fold (Figure 9c: dissociation constant, K_D_ = 1.5 µM, IC_50_ = 4.0 µM), predominantly by pre-organizing the SLe^x^-core with a novel GlcNAc mimetic and leaving the pharmacophores as present in native SLe^x^ [110].

Ernst and co-workers further optimized E-selectin antagonists using fragment based discovery techniques to select ligands able to bind in a second site near the sLe^x^ mimic binding site [112]. Fragments were screened for E-selectin binding using spin-lock filtered NMR experiments. The hits were then retested in the presence of the first site ligand which was labeled with a spin-label probe. This then enabled the identification of fragments which were binding in the vicinity of the spin-label via paramagnetic relaxation enhancement spectroscopy. These fragments were then connected to the sLe^x^ mimic through flexible linkers of variable length and tested by SPR for interaction with E-selectin. The most potent antagonists so obtained had K_D_ values between 30 and 89 nM and a half-life of the ligand protein complex in the range 4–5 min (e.g., Figure 9d: K_D_ = 30 nM, t_1/2_ = 4.1 min). This was a substantial improvement with respect to affinity and half-life as carbohydrate-lectin interactions are generally characterized by micro- to millimolar affinities and half-lives in the seconds [113].

**Figure 10:**
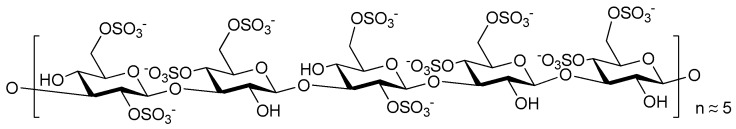
Structure of PS3; the sodium salt of a β-1,3-glucan sulfate with a degree of sulfation of 2.2 and a polydispersity of 25 corresponding to a mean molecular weight of 10,000.

Others have also developed heparin mimetics that target the selectins [114]. Some of these were produced by varying the degree of sulfation of natural linear glucans with varying degrees of polymerization and different linkages between their monosaccharide units. This work revealed that charge density (degree of sulfation), rather than molecular weight is the more important factor in determining whether the various sulfated glucans inhibit P-selectin activity. The monomer glycosidic linkage also seemed to play a role, as mimetics of comparable length and degree of sulfation but with a backbone of β-1,3-linked glucose units, were inferior to mimetics with a backbone consisting of α-1,4/1,6-glucans. The increased activity was attributed to the greater flexibility of the latter mimetics. A kinetic study, on the binding interactions of some of these compounds with P-selectin, was quite informative [115]. This work revealed that mimetic occupancy time on P-selectin was critical for inhibitory activity, and mimetics with a disassociation rate markedly slower than the natural ligand should be good inhibitors. One of these compounds was PS3 (Figure 10), a low polydispersity β-1,3-glucan sulfate in which the primary hydroxyl group at position 6 was fully sulfated, the secondary hydroxyl groups at positions 2 and 4 were equally sulfated to about 60%, giving an overall degree of sulfation of 2.2 sulfates per glucose unit, and the degree of polymerization was about 25. This compound significantly inhibited peripheral blood mononuclear cell interactions with endothelial cells under flow conditions, an effect attributed to its inhibition of both L-selectin and P-selectin, but not E-selectin [116]. It was also shown to act as an anti-inflammatory agent in a murine model of contact hypersensitivity, but it’s in vivo potency was not investigated at concentrations other than 25 mg kg^−1^, which is quite a high dose.

**Figure 11:**
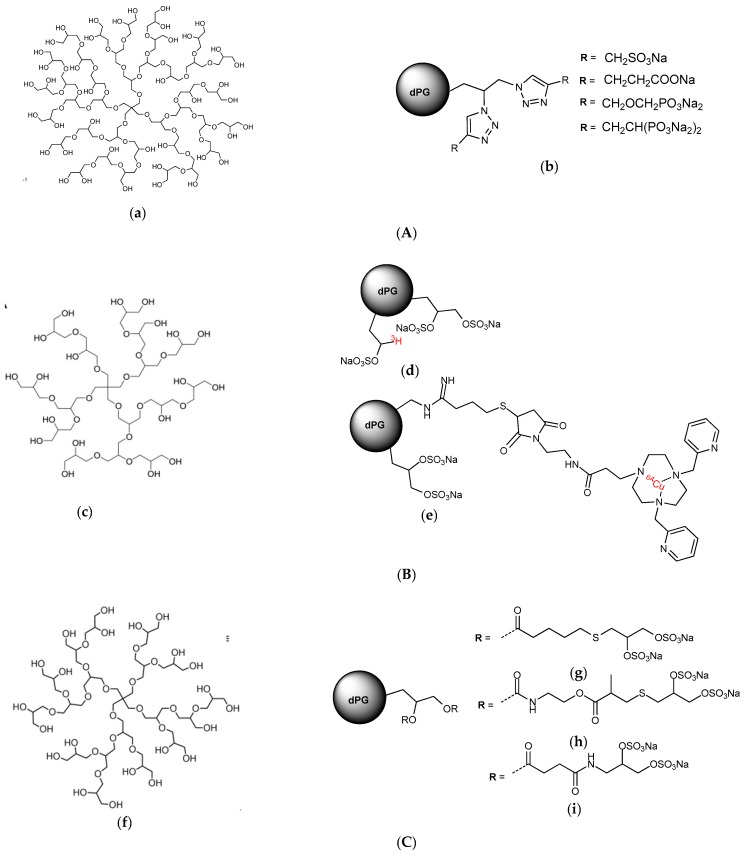
Structure of dendritic polyglycerol heparin mimetics: The structure of dendritic polyglycerols (dPG) scaffold illustrates an idealized fragment of the polymer. (**A**) Structure of (**a**) dendritic polyglycerol starting material and (**b**) dendritic polyglycerol heparin mimetics synthesized via click coupling by Weinhart et al. [117]; (**B**) Structure of (**c**) dendritic polyglycerol starting material and (**d**,**e**) radiolabeled dendritic polyglycerol heparin mimetics synthesized by Pant et al. [118]; (**C**) Structure of (**f**) dendritic polyglycerol starting material and (**g**–**i**) shell cleavable dendritic polyglycerol heparin mimetics synthesized by Reimann et al. [95].

The notion that greater potency against the selectins may be achieved by multivalent, dendritic compounds of polyglycerol anions has also been investigated. A variety of different compounds were made using dendritic polyglycerols of different molecular weights as the starting material. The dendritic polyglycerols were synthesized by one-step anionic ring-opening multibranching polymerization of glycidol on a polyol initiator. These compounds were functionalized with different polyanions with a high degree of functionalization (>80%) using click chemistry (Figure 11A). Those with a core size of 6000 Da and functionalized with sulfates most effectively inhibited L-selectin with low nanomolar range IC_50_ values [117]. Importantly, the in vivo pharmacokinetics of the best of these compounds has also been examined via radiolabeling. The radiolabeling of the dendritic polyglycerol candidate was accomplished by an oxidation-reduction process with sodium periodate and [^3^H]-borohydride followed by sulfation using SO_3_.pyridine complex. This method represents a mild straight forward labelling technique for the introduction of titrium into 1,2-diol containing molecules without the use of radioactive monomers. An optimized radiochemical yield of >80% was achieved for this reaction, proving its efficacy. ^64^Cu-labeled compounds were synthesized by conjugating Cu(II) chelators to the partially aminated dendrimer (Figure 11B). A radiolabelling yield of >99% was achieved within 5 min with a high radiochemical purity, eliminating the need for further purification [118]. These radiolabelling methods are likely to be applicable to other heparin mimetics as they move into the pre-clinical phase. In this case, the biodistribution studies in healthy mice and rats revealed that the polysulfated dendrimers accumulated in the liver and spleen and although small amounts were excreted via the kidneys there was still evidence of organ accumulation after 3 weeks, which was not a favorable distribution pattern for a drug.

To overcome this problem shell cleavable polysulfated dendrimers were synthesized and investigated for competitive L-selectin binding, blood coagulation, complement activation and degradation in vitro. This synthesis and the biological activities are described in the report by Reimann et al. [95]. This work revealed that two of these compounds dPG-thioglyceryl pentanoatyl sulfate (Figure 11C(h)) and pPG-thioglyceryl methylpropanoatyl sulfate (Figure 11C(g)) containing long flexible hydrophobic linkers which included respectively, either an ester functionality, or a carbamate and an ester functionality, had potent complement pathway inhibition activity, minimal anticoagulant activity and high picomolar IC_50_ values in the L-selectin binding assay. These two compounds also degraded quite readily and so were considered as interesting compounds for long term treatment of chronic inflammation or as a new class of anti-complement therapeutic for preventing tissue damage within inflammatory disease [95].

Heparin and HS are known to interact with numerous proteins in all three pathways of the complement cascade. Most of these interactions have a regulatory role and most result in the inhibition of the complement cascade [97]. Structure-activity relationships of more than 40 structurally distinct sulfated glycans with the serpin C1 inhibitor (C1-INH) and the serine protease, C1s, have been examined [119]. C1-INH inactivates the initiating enzymes of the complement classical pathway, C1r and C1s, as well as the initiating serine proteases of the mannose-binding lectin pathway, Mannose binding lectin Associated Serine Protease (MASP)-1 and MASP-2. C1-INH is also the most important inhibitor of the plasma proteases kallikrein, Factor XIa and Factor XIIa of the intrinsic coagulation pathway. It was found that the sulfated glycans potentiated C1-INH activity and shortened the time for C1s inhibition by about 50% and this potentiating effect was dependent on degree of sulfation and molecular weight, but glycan structure was also important. The linear β-1,3-glucan structure was favored over the branched α-1,6-glucan structure of dextrans, and the former structure was also favored over heparin. The dependence on degree of sulfation and size was linked so that a small compound like the pentasaccharide Fondaparinux, which is highly sulfated, was slightly potentiating, whereas if the degree of sulfation was low, a much larger sulfated glycan was required for any C1-INH potentiation. It is believed that the polyanion links the basic surfaces of C1s and C1-INH like the filling in a protein sandwich [120], which is different from the “bridging” mechanism of antithrombin III and thrombin whereby these two proteins bind adjacent regions on a single heparin chain. It could be argued that as the entire “top” face of C1-INH is positively charged, longer polyanions of low sulfation are required for charge neutralization of this face on C1-INH and trimer formation. Schoenfeld et al., used this rational, plus the argument that C1-INH amplifiers should have both: low anticoagulant activity, and the improved pharmacokinetics of a rather low molecular size, to promote the β-1,3-glucan sulfates as promising candidates for further investigation [119].

Additionally, a number of GAG analogues that bind to chemokines are under development as novel anti-inflammatory drugs. In one study compounds were developed based on two different approaches. Firstly, a structure based approach was used after initial screening of potential small molecule binders using protein NMR on a target chemokine, in this case CCL5 (or RANTES) [96]. Two small molecules shown to bind to separate sites on CCL5 by NMR and X-ray crystallography were linked to form a chimera, the hope being that the chimera would have better anti-inflammatory activity than the separate molecules. In the second approach by the same group, commercially available short oligosaccharides were persulfated. In vitro, the molecules prevented chemokine-GAG binding and chemokine receptor activation without disrupting coagulation. However, in vivo variable results were seen in a murine peritoneal recruitment assay, with the chimeric molecule enhancing cell recruitment in this assay [96]. In this study, the crystallography was performed at low pH. However, our work with CCL5 revealed that the sites where heparin tetrasaccharides bind this chemokine vary depending on the pH [15]. Thus, the crystallography data would have indicated where the heparin mimetics bound CCL5 at low pH (pH 3.5), rather than where they bind at the pH encountered in vivo, and as this was unlikely to have been the same site the structural data may have provided misleading information. Nevertheless, some of the persulfated oligosaccharides were shown to inhibit inflammation in a delayed-type hypersensitivity model and in an antigen-induced arthritis model [96], the reasons for the different activities in the different models is unknown.

Other workers have examined heparin mimicking glycopolymers for their ability to bind CCL5. The rational was that a HS disaccharide epitope presented as a multivalent, pendant array on a polynorbornene backbone might antagonize CCL5 if the binding affinity was enhanced by increased avidity [121]. Controlling the sulfation pattern before polymerization allowed the production of four different glycopolymers each with a different pattern of sulfation on the disaccharide. These sulfation patterns were: 2-*O*-sulfated IdoA 1,4-linked to *N*- and 6-*O*-sulfated GlcN; 2-*O*-sulfated IdoA 1,4-linked to *N*-acetylated, 6-*O*-sulfated GlcN; IdoA 1,4-linked to *N*-acetylated GlcN, and IdoA 1,4-linked to *N*- and 6-*O*-sulfated GlcN. The resulting mimetics were variations on the structures shown in Figure 4. Of these mimetics the glycopolymer carrying the trisulfated disaccharide most effectively competed with heparin for binding CCL5, and not surprisingly, the non-sulfated glycopolymer was without activity. Chemotactic activity mediated by CCL5 binding its receptor CCR3 was also inhibited by both heparin and trisulfated disaccharide glycopolymer, but chemotaxis of cells bearing the alternative CCL5 receptor, CCR5, was not inhibited. Unfortunately the efficacy of this glycopolymer in an in vivo inflammation model was not reported, nor is it clear how the glycopolymer would bind CCL5 oligomers.

**Figure 12:**
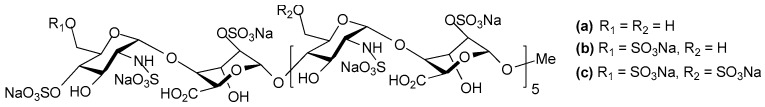
Site-specifically 6-*O*-sulfated dodecasaccharides: (**a**) completely non-6-*O*-sulfated (**b**) site-selectively mono-6-*O*-sulfated (**c**) fully 6-*O*-sulfated.

CCL5 is not the only chemokine that has been studied for its interaction with heparin mimetics. A recent study using synthetic heparin/HS-like dodecasaccharides has revealed that that addition of a single 6-*O*-sulfate to the glucosamine at the non-reducing terminus of the mimetic alters which chemokine binds [122]. The use of the 3 homogeneous, synthetic dodecasaccharides of known structures indicated that the presence or absence of this site-specific 6-*O*-sulfation determined whether the signaling of either of the chemokines CXCL8 (or IL-8) or CXCL12 was inhibited. The dodecasaccharides were of the following structures: (1) non-reducing terminal *N*- and 4-*O*-sulfated GlcN 1,4 linked 2-*O*-sulfated IdoA 1,4 linked [*N*-sulfated GlcN 1,4-linked 2-*O*-sulfated IdoA]_5_ (Figure 12a); (**2**) non-reducing terminal *N*-sulfated,4,6-*O*-sulfated GlcN 1,4-linked 2-*O*-sulfated IdoA 1,4-linked [*N*-sulfated GlcN 1,4-linked 2-*O*-sulfated IdoA]_5_ (Figure 12b); and (3) fully 6-*O*-sulfated dodecasaccharide (Figure 12c). Interestingly the fully 6-*O*-sulfated dodecasaccharide did not preferentially inhibit either CXCL8, or CXCL12, but its biological activity resembled that of a native dp12 arising from digesting commercial heparin. The mimetics also demonstrated these behaviors in in vivo models. For example, analogue **a** (see Figure 12) inhibited CXCL8 induced neutrophil infiltration whereas analogue **b** (see Figure 12) with the reducing end 6-*O*-sulfate GlcN did not, and analogue **b** inhibited CXCL12 induced macrophage infiltration whereas analogue **a,** which lacked the terminal 6-*O*-sulfate moiety did not. These data indicated that for these chemokines the overall sulfation level of the dodecasaccharide was not the critical feature but rather the single non-reducing end 6-*O*-sulfate GlcN determined the biological behavior. The study authors comment that their findings suggest that, “other significant site-specific sulfation-determined effects await discovery and biomedical exploitation” [122].

**Figure 13:**
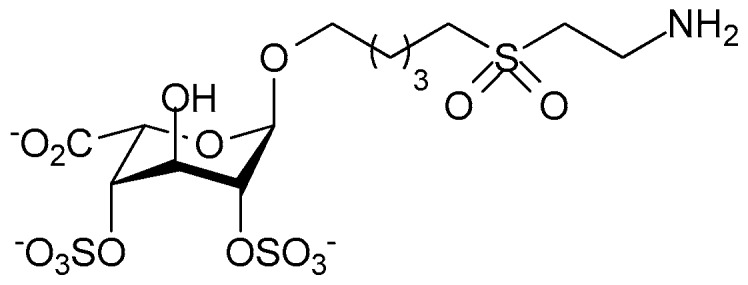
The unnatural synthetic monosaccharide, Di-S-IdoA containing two axial sulfate groups.

Homogeneous, discrete GAG-mimetics are proving to be powerful tools for unravelling the structure-specificity issues that have been haunting GAG biology for decades. A glycan array of heparin-like oligosaccharides varying from monomer to hexamer was used to probe the binding specificity of the chemokine, CCL20 [123]. This chemokine bound the un-natural, synthetic monosaccharide 2,4-*O*-sulfated IdoA (Figure 13) with micromolar affinity, and this di-sulfated IdoA interfered with CCL20 heparan sulfate binding in a concentration dependent fashion and it also inhibited the binding of CXCL8 and L-selectin. The in vivo effects of di-sulfated IdoA in a murine model of allergy were intriguing. Injection of di-sulfated IdoA immediately prior to allergen challenge decreased mucus secretion and T lymphocyte recruitment into the lungs. Administering di-sulfated IdoA by inhalation before allergen challenge decreased CCL20 staining on lung endothelial cells and lymphocyte recruitment, as measured by lymphocyte numbers in bronchi alveolar lavage fluid (BALF). These data were interpreted as suggesting that CCL20-mediated recruitment of Th17 and Th2 lymphocytes were critical for the early stages of the asthmatic response. The di-sulfated IdoA was said to act by binding to CCL20, thereby preventing CCL20 from accumulating on HS on the airway endothelium and from binding its receptor CCR6 on the lymphocytes and triggering their recruitment. Importantly, other chemokines (CCL19, CCL21, CCL25, CCL28, CXCL12, CXCL13 and CXCL16) did not bind 2,4-*O*-sulfated IdoA indicating the specificity of the response [124].

**Figure 14:**
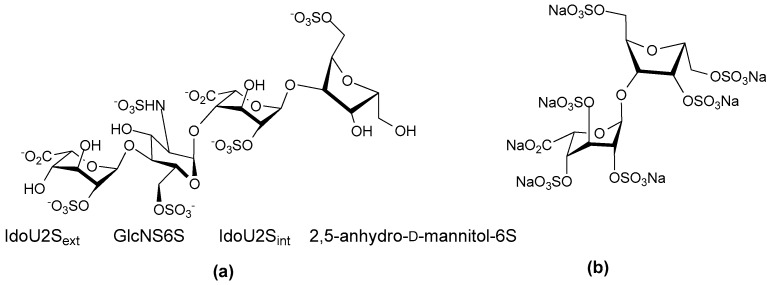
(**a**) Heparin tetrasaccharide which possess the minimal chain length for anti-allergic and anti-inflammatory properties (**b**) Supersulfated heparin derived disaccharide, Hep-SSD.

Abraham and co-workers similarly tested a disaccharide heparin analogue in an animal asthma model. They first described, what in their view is, a minimal chain length and structural sequence of the anti-allergic domain of heparin. In this study, it was demonstrated that the anti-allergic activity of heparin resided in a tetrasccharide sequence and that the domain responsible for anticoagulant and anti-allergic activity of heparin were distinctly different (Figure 14a) [125]. Later, Abraham and co-workers also demonstrated that “supersulfation” of an anticoagulation inactive disaccharide fragment conferred anti-allergic activity. The disaccharide Hep-SSD (Figure 14b) was prepared by nitrous acid depolymerization of porcine intestinal heparin followed by size exclusion chromatography. The disaccharide fragment was then sulfated to obtain supersulfated Hep-SSD. This disaccharide, Hep-SSD was shown to inhibit allergic airway responses in sheep model of asthma and it displayed activity by both aerosol and oral routes [126]. There has been considerable interest over a number of years in the possibility that non-anticoagulant heparin derivatives may be useful anti-inflammatories for the management of asthma and a number of small clinical trials have examined this notion (see review: [127]). However, to our knowledge this interest has yet to extend to testing heparin mimetics in clinical trials as anti-asthmatics despite the promising data from animal studies.

**Figure 15:**
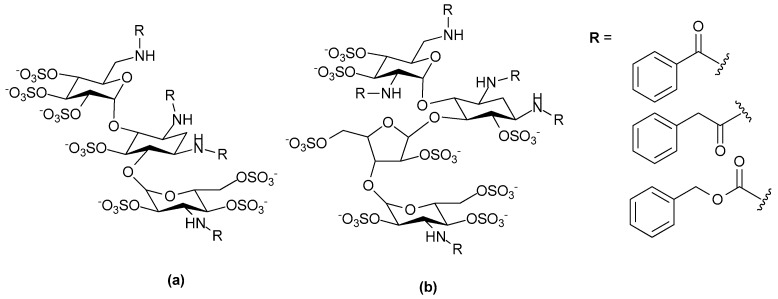
Structure and degree of sulfation of *N*-arylacyl *O*-sulfonated aminoglycosides (**a**) Kanamycin core derivatives (**b**) Neomycin core derivatives.

Another lung disease in which heparin mimetics may exert useful anti-inflammatory effects is chronic obstructive pulmonary disease (COPD). This is a disease of neutrophil infiltration and much of the tissue damage is caused by serine proteases released from neutrophil granules. These include neutrophil elastase, cathepsin G and proteinase 3, enzymes known to be inhibited by heparin. Against this background Craciun et al. synthesized, structurally characterized, and tested a panel of of *N*-arylacyl *O*-sulfonated aminoglycosides to identify inhibitors of these neutrophil serine proteases [128]. This work identified a kanamycin- and a neomycin-based compound as compounds of interest; the kanamycin-based compound (Figure 15a) inhibited all three enzymes whereas the neomycin-based compound (Figure 15b) inhibited neutrophil elastase and cathepsin G. Further functional optimization of the kanamycin-based compound is continuing with the view of developing an inhaled drug for attenuation of protease-mediated lung disease. Others have produced promising compounds using heparin as the starting material. For example, Fryer et al. reported the modification of unfractionated heparin to yield a 2,3-*O*-desulfated heparin (ODSH) that lacks anticoagulant activity, but retains the anti-inflammatory characteristics of the parent molecule [129]. ODSH was developed by selective desulfation of unmodified heparin under extreme alkaline conditions [130]. In preclinical studies, ODSH has shown promising anti-inflammatory activity by inhibiting airway hyperactivity and airway smooth muscle proliferation in mammals. Clinical trials of ODSH in patients with exacerbations of COPD are ongoing [131].

Clearly, heparin mimetics could be a novel class of therapeutic compounds for a range of inflammatory diseases. However, further work is required to unravel the mechanisms of action that give rise to the anti-inflammatory effects of these molecules. The protein-binding specificities of the mimetics studied to date suggest that different mimetic structures will be optimal for inflammatory diseases with different etiologies. However, it is also likely that the pleiotropic effects of heparin mimetics is a factor in their in vivo efficacy as anti-inflammatory agents. The optimization of drug candidates by increasing their binding affinities for particular protein targets should take into account the likely requirement for pleiotropic activities in an effective anti-inflammation drug. Nevertheless, the results of an increasing number of studies support the likelihood that it will be possible to design agents, mimicking heparin, that exploit the anti-inflammatory actions of the parent molecule but lack its anticoagulation activity.

## 6. Heparin Mimetics: Potential Toxicities

The therapeutic potential of heparin mimetics is dependent on any drug induced adverse effects, and there are well known molecular interactions that could be undesirable depending on the disease indication and the proteins being targeted. Although in animal studies these types of molecules are generally well tolerated there are some exceptions. For example, at high concentrations (25 and 75 mg/kg intraperitoneally twice a week) pentosan polysulfate, a sulfated xylan polymer, caused immediate mortality in 60% of the mice under study [132]. Anticoagulation was assumed to be a contributor to the adverse effect of pentosan polysulfate in this study, and it is the most obvious possible undesirable effect, if the mimetic is targeting a disease which does not have coagulation as a component of its etiology.

A number of different approaches have been taken to reduce the anticoagulant activities of heparin derivatives and heparin mimetics. Casu and his colleagues pioneered the “glycol-splitting” of heparin chains, where the C(2)-C(3) bond of an un-sulfated uronic acid is split by periodate oxidation with subsequent borohydride reduction [133]. The glucuronic acid in the antithrombin III binding motif is cleaved by this reaction and as this residue is essential for high affinity antithrombin III binding these glycol-split heparin chains have very low anticoagulant activity, but retain the other main activities of heparin. The extent of glycol-splitting can be varied by controlled partial 2-*O*-desulfation followed by periodate oxidation/borohydride reduction to give regions of pentasulfated trisaccharides adjacent to a glycol-split uronic acid. Through this approach some activities of heparin such as FGF-2 binding were retained, whereas others were reduced (e.g., induction of FGF-2 dimerization [134]), and the pattern of activities was found to vary depending on the extent of glycol-splitting. The increased conformational flexibility that is introduced by the more freely-rotating glycol-split uronic acids contributes to the variability in activity. A number of different non-anticoagulant, glycol-split heparins have been produced that differ in molecular weight, extent of glycol-splitting and activities (see the recent review [135]).

Although heparin mimetics that do not resemble the pentasaccharide motif in heparin that is required for high affinity binding to antithrombin III may not be expected to have anticoagulant activity, or to have very low anticoagulant activity, this may not always be the case. For example, sulfate glycans from some marine organisms have anticoagulant activity and their mode of action is enhancement of the antithrombin III and heparin cofactor II inhibitory activities, but the major glycosylation and sulfation structures of these glycans were found not to resemble the antithrombin III motif that is present in heparin [136]. There are numerous examples of non-mammalian sulfated glycan structures with anticoagulant activity that can be primarily attributed to antithrombin III and /or heparin cofactor II inhibition of thrombin, such that it has been possible to propose the types of sulfated glycan structures that are required for anticoagulant activity [137]. Moreover, as well as pentosan polysulfate other synthetic heparin mimetics have been produced that interact with antithrombin III or heparin cofactor II and hence act as anticoagulants, these include a polysulfated trehalose [138] and a sulfated mannogalactan [139]. The interaction of heparin cofactor II with heparin is considered to resemble the interaction of antithrombin III with low affinity heparin (i.e., heparin that does not have the antithrombin III-binding pentasaccharide), with affinity being determined by ionic strength and heparin size [140]. Given this, it is not surprising that heparin mimetics may interact with one or both of these proteins and inhibit thrombin. Thus, anticoagulation testing should be included in the toxicity analyses of all heparin mimetics designed for non-anticoagulation indications. It is also conceivable that a heparin mimetic could contribute to anticoagulation by displacing tissue factor pathway inhibitor (TFPI) from the vascular endothelium and into the circulation. Thus, an in vivo assessment of anticoagulation effects of heparin mimetics in an animal model where TFPI is produced by endothelial cells and is bound by the vascular endothelium, as it is in humans [141], should be part of the toxicity package of a mimetic with planned intravenous delivery.

Other factors in the coagulation cascade may also react with heparin mimetics and of particular interest is the serine protease factor XII (FXII). When FXII comes into contact with negatively charged surfaces this triggers a conformational change in the protein to give activated FXII (FXIIa). FXIIa triggers the proinflammatory and procoagulant pathways of the contact system (the reader is referred to recent reviews for details, [142]). Briefly, FXIIa cleavage of plasma prekallikrein gives plasma kallikrein which reciprocally activates FXII, and results in vastly more FXIIa than originally produced by the negatively charged surface. Plasma kallikrein cleaves high molecular weight kininogen to release bradykinin, a potent proinflammatory peptide. Plasma kallikrein also converts plasminogen into plasmin which is involved in fibrin degradation. FXIIa activates the classical complement pathway (via C1r and C1s) resulting in production of the anaphyatoxins C3a and C5a which stimulate inflammation. Finally, the intrinsic coagulation cascade is activated by FXIIa cleavage of FXI giving FXIa, which initiates a series of cleavage reactions that generate thrombin, fibrin formation and fibrin clots.

A number of natural negatively charged substances have the potential to trigger FXII activation, these include heparin, heparan sulfate, chondroitin sulfates, dermatan sulfates, lipoproteins, platelet polyphosphate, l-homocysteine and so on [142,143], but some artificial substances including 500 kDa dextran sulfate and chemically over sulfated glycosaminoglycans are particularly potent FXII activators [144]. A comparison of the various oversulfated glycosaminoglycans suggested that activation of the contact system is “pattern-dependent” rather than “structure-dependent” and the degree of chemical sulfation is a key determinant as to whether kallikrein activity is induced in human plasma. The importance of assessing if a heparin mimetic designed for therapeutic application activates the contact system is highlighted by what became known as the “heparin contamination crisis” that occurred in 2007 and 2008. Over sulfated chondroitin sulfate (OSCS) contamination of heparin was discovered to be the main causative agent. Activation of both the kinin-kallikrein system and the complement pathway (evident by the levels of C3a and C5a generated), via activation of FXIIa, were deemed responsible for the adverse effects suffered by patients [145], and which potentially led to the 246 recorded deaths of patients receiving heparin during that period [146]. The enhanced production of bradykinin is believed to be responsible for the hypotension and angioedema experienced by around 50% of patients who suffered adverse events. It is likely that route of delivery, and the dose of the contaminated heparin delivered were factors contributing to the severity of the adverse events. These conclusions are supported by a rat study in which the hypotensive effects experienced by patients could be replicated in rats by intravenous delivery of OSCS-contaminated heparin, whereas subcutaneous administration was not effective, moreover, the hypotensive effect was dependent upon the concentration of the contaminating OSCS [147].

Further studies revealed OSCS interacts with some contact system and coagulation system components in different ways from heparin [148]. Firstly, the anticoagulant activity of OSCS is primarily through heparin cofactor II mediated inhibition of thrombin. Although OSCS binds tightly to antithrombin III, this binding does not cause the conformational change in antithrombin III that is required for its inactivation of thrombin and FXa, moreover, OSCS does not compete with heparin for binding to antithrombin III. Secondly, unlike heparin, OSCS binds tightly to FXIIa. These activity differences may be useful indicators for distinguishing whether a heparin mimetic has OSCS-like toxicology. The generation of the complement pathway anaphylatoxins C3a and C5a by OSCS was particularly intriguing as this appeared to occur via a mechanism that did not involve C3 and C5 convertases. Rather kallikrein induced by FXIIa appeared to be the key initiating factor [145]. When screening for contact system activation and high kallikrein activities the choice of plasma is important as animal plasmas have been reported to differ according to species in their capacity to give rise to the high kallikrein activities seen with human plasma following stimulation with OSCS [145,147]. Curiously a veterinary drug, polysulfated glycosaminoglycan (PSGAG) marketed as Adequan^®^, also an oversulfated chondroitin sulfate, is administered intramuscularly for animal (dog and horse) osteoarthritis. The mode of delivery is likely to limit FXIIa levels and the kallikrein effect in the animals treated, but this drug has been shown to resemble OSCS in its reactivity with some complement pathway proteins and may activate FXII if delivered by an intravascular route [149].

An adverse event that could be triggered by heparin mimetics is that of drug induced thrombocytopenia. By analogy with heparin induced thrombocytopenia (HIT), this could occur if the mimetic binds PF4 and so exposes antigenic epitopes on PF4 which triggers an antibody response to the mimetic-PF4 complex and the formation of large immune complexes. Antibodies in these complexes bind Fc-receptors on platelets and monocytes triggering platelet aggregation, activation and the release of pro-thrombotic mediators which markedly increase the risk of thrombosis. Thrombocytopenia (a drop in platelet count of around 50% or more) results from the clearance of platelet aggregates. In most HIT patients thrombotic complications and thrombocytopenia occur concurrently, although thrombosis is the main contributor to disease and mortality [150]. Clinical reports have indicated pentosan polysulfate can induce thrombocytopenia and thrombosis at least to the same degree as heparin [151], and in vitro studies have shown that polyphosphates form antigenic complexes with PF4 which are recognized by anti-PF4 antibodies [48]. Thus, neither the monosaccharide composition nor the structure of the anion are determining features for PF4 binding. Studies with heparin showed that ≥10 saccharides and >13 sulfate groups are required for heparin fragments to bind PF4, trigger neoepitope exposure and HIT antibody binding, and it was concluded that charge density and oligosaccharide size were more important than a specific sulfation pattern for PF4 binding [152]. Given these findings it is probable heparin mimetics that broadly fit the criteria of ≥10 saccharides and >13 anions have the potential to bind PF4 in such a way so as to trigger drug induced thrombocytopenia. However, the clinical data obtained with PI-88 indicated that heparin mimetics smaller than 10 saccharides may also trigger immune mediated thrombocytopenia with some patients developing HIT-like disease [69]. Clearly PF4 binding should be included in the off-target screening program for heparin mimetics.

## 7. Conclusions

Decades of research have highlighted the enormous potential of heparin mimetics as drugs. The rationale for developing heparin mimetic drugs as opposed to using native heparin, or heparin oligosaccharides as drugs, in part depends on the disease being targeted. However, regardless of disease indication, there is a move away from heterogeneous natural product drugs derived from animal tissues, towards homogeneous, synthetic or semi-synthetic, structurally defined entities. The heparin contamination crisis of 2007 and 2008 highlighted potential problems that can arise with heterogeneous natural product drugs, and served to provide an additional impetus to heparin mimetic development. As with all drug discovery programs increased potency, increased selectivity and better pharmacokinetics are also goals of heparin mimetic development. Although the LMWHs have improved bioavailability and pharmacokinetic profiles over that of the parent product, they retain the issues associated with heterogeneity and natural product origins. Heparin oligosaccharides of uniform length and structure isolated from heparin are not viable drugs in our view. It is possible, using the appropriate purification strategies, to obtain pure oligosaccharide structures [14,15], but the final yields are so poor as to be prohibitive for a drug. An alternative would be the production of synthetic heparin oligosaccharides of defined structure; the marketing of Fondaparinux indicates this is possible. However, as Fondaparinux synthesis requires around 50 chemical steps other avenues to achieve heparin structures with less steps are being explored, and encouraging advances in chemoenzymatic methods are being achieved [153].

The heparin mimetic approach is different, in that exact copies of natural heparin oligosaccharides are not the goal, rather ease of synthesis, homogeneity (usually) and a reduction in the promiscuity of heparin’s protein binding behavior are the usual goals. In the examples discussed here the structural diversity of heparin mimetics is evident; some display only very minor differences from the natural product, whereas other mimetics bear no resemblance to heparin other than that they are polyanionic and they inhibit heparin from binding to their protein target(s). In the design of heparin mimetics there is the opportunity to tailor the mimetic structure to favor some of heparin’s functions and eliminate others. For example, many of the mimetics targeting cancer or inflammation have been designed so they cannot bind antithrombin III, and so anticoagulation activity can only occur by other pathways. In contrast, anticoagulant mimetics can be designed to bind antithrombin III but not PF4, thereby removing drug induced thrombocytopenia as a potential side-effect (see this review). Heparin mimetics generally are less heterogeneous than heparin, or are homogeneous, and thus they may be expected to display a greater specificity and potency of action than heparin. Detailed structural studies of heparin/GAG-protein interactions using NMR spectroscopy, X-ray crystallography and molecular modelling techniques have greatly aided the design of heparin mimetics. However, to be effective the conditions of these in vitro binding studies should mirror, as much as possible, the conditions likely to be encountered in vivo, as the tissue microenvironment pH and cation composition may contribute to the strength of mimetic binding and position on the protein where the mimetic binds.

The consequences of possible pleiotropic effects of heparin mimetics will vary depending on the disease indication. The three disease areas discussed here ranged from the effects of a clear biochemical pathway (blood coagulation and thrombosis), to complex disease processes (cancer and inflammation) that involve a multitude of different molecular pathways, or binding events, a number of which involve heparin/HS-protein interactions. Exquisite specificity of anti-coagulant heparin mimetics would be advantageous, whereas exquisite specificity for one protein target may not provide the therapeutic outcome that is desired in the other two disease indications. In inflammatory diseases and cancers, the molecular redundancy of the pathology is such that although one pathway may be blocked the disease can still progress. There is also considerable overlap in the disease processes, for example thrombogenicity is often associated with cancer or inflammation. Hence, mimetics that display a degree of polypharmacy are likely to be of more therapeutic benefit than a mimetic that is directed towards a single target. The challenge will be to maximize the benefits of polypharmacy whilst managing any adverse effects that could arise from pleiotropic binding behavior. Drug delivery regimes that largely restrict the drug to the site of disease is one way of managing this issue, although this may not always be possible in the case of cancers. Nevertheless, this field is advancing rapidly as new synthetic approaches are explored alongside the advances in structural analysis techniques that have occurred in recent years. As a consequence, we believe heparin mimetics with superior absorption, distribution, metabolism and excretion properties will be developed and for at least some of these mimetics significant therapeutic benefits will result from their ability to act in multiple molecular pathways.
